# Beta-Cell Age Calculator, a Translational Yardstick to Communicate Diabetes Risk with Patients: Tehran Lipid and Glucose Study

**DOI:** 10.5402/2013/541091

**Published:** 2012-12-02

**Authors:** Mohammadreza Bozorgmanesh, Farzad Hadaegh, Fereidoun Azizi

**Affiliations:** ^1^Prevention of Metabolic Disorders Research Center, Research Institute for Endocrine Sciences (RIES), Shahid Beheshti University of Medical Sciences, P.O. Box 19395-4763, Tehran 1985717413, Iran; ^2^Endocrine Research Center, Research Institute for Endocrine Sciences (RIES), Shahid Beheshti University of Medical Sciences, Tehran 1985717413, Iran

## Abstract

*Aims*. To provide a yardstick for physicians/patients to efficiently communicate/measure incident diabetes risk. *Methods*. We included data on 5,960 (3,438 women) diabetes-free adults, aged ≥20 years at baseline who either developed diabetes during two consecutive examinations or completed the followup. Age, systolic blood pressure, family history of diabetes, waist-to-height ratio (WHtR), triglyceride-to-high-density lipoprotein cholesterol ratio (TG/HDLD-C), and fasting plasma glucose (FPG) were introduced into an accelerated failure time regression model. *Results*. Annual diabetes incidence rate was 0.85/1000-person (95% CIs 0.77–0.94). Point-score-system incorporated age (1 point for >65 years), family history of diabetes (4 points), systolic blood pressure (−1 to 3 points), WHtR (−4 to 6 points), TG/HDL-C (1 point for ≥1.5), and FPG (0 to 27 points). Harrell's *C* statistic = 0.830 (95% CIs 0.808–0.852) and Hosmer-Lemeshow *χ* ^2^ = 9.7 (*P* for lack of fitness = 0.462) indicated good discrimination and calibration. We defined beta-cell age as chronological age of a person with the same predicted risk but all risk factors at the normal levels (i.e., WHtR 0.50, no family history of diabetes, Ln (TG/HDL-C) = 0.531, and FPG = 4.9 (mmol*·*L^−1^)). *Conclusion*. Hereby, we have made it also possible to estimate wide ranges of “beta-cell age” for most chronological ages to assist clinician with risk communication.

## 1. Introduction

Type 2 diabetes (hereafter diabetes) is a common and serious condition associated with reduced life expectancy and microvascular and macrovascular worldwide morbidity [1–4]. Clinical trials have shown that diabetes can be prevented or the onset delayed by lifestyle modifications more efficiently than by pharmaceutical [5–10]. Our ability to predict and prevent diabetes in the general population is limited; lifestyle modification programs may entail substantial costs and medication interventions incur costs and may cause harm, which may outweigh benefits when these interventions are applied to individuals at relatively low risk for diabetes.

We developed and published the first prediction model based on the data from the Tehran Lipid and Glucose Study (TLGS) and demonstrated that complex models are not needed to predict diabetes [11]. However, years after introducing the TLGS absolute risk-based recommendations, our experience suggests that absolute risk is still not well understood by many practitioners or patients. Furthermore, it has been observed that even when provided with risk calculation tools, clinicians sometimes apply them inappropriately [12, 13]. Someone's behaviors constituting their current lifestyle are deep-rooted in their cognition and feeling regarding diabetes risk. Changing current behaviors, thus, mandates change in feeling and the feeling in turn has been shown to be subjected to change as cognition changes [14]. Here is the vantage position for diabetes risk communication to come forward: motivational interview [15].

In this paper, therefore, we updated the TLGS diabetes prediction model [11] by deploying survival regression and provided a method for translating the predicted risk to a “beta-cell age” (i.e., the age of a person with the same predicted 6-year risk but with all normal risk factor levels) to improve risk communication. 

## 2. Research Design and Methods

### 2.1. Study Population

**Figure 1:**
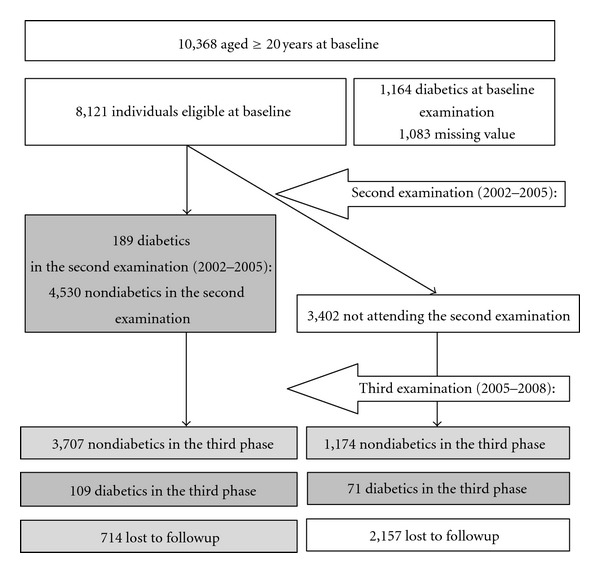
Study sample, inclusion, and exclusions.

The Tehran Lipid and Glucose Study (TLGS) is a prospective population-based study performed on a representative sample of the Tehran population, with the aim of determining the prevalence of noncommunicable disease risk factors and developing a healthy lifestyle to improve them. The baseline survey was performed from February 1999 to July 2001 (the first examination). After this cross-sectional phase, individuals were assigned to a prospective study with follow-up examinations on a triennial basis. For the current analysis, we used the data from 10,368 individuals older than 20 years of age attending the baseline examination. Participants with prevalent diabetes (*n* = 1,164), and those whose diabetes status could not be ascertained (*n* = 989) or those with missing data on potential predictors (*n* = 1,731) were excluded (figures may overlap). After these exclusions, there were 8,121 participants free of diabetes at baseline. In the current analyses, we included data on 5,960 (3,438 women) individuals who attended at least one of two of follow-up examinations; one from September 2001 to August 2005 (the second examination) and the other from April 2005 to March 2008 (the third examination) (Figure 1).

### 2.2. Clinical and Laboratory Measurements

A trained interviewer collected information using a pretested questionnaire. The information obtained included demographic data, family history of diabetes, and drug use. Two measurements of systolic blood pressure (SBP) and diastolic blood pressure (DBP) were taken using a standardized mercury sphygmomanometer on the right arm, after a 15-minute rest in a sitting position; mean of the two measurements was considered as the subject's blood pressure [12]. Weight was measured, with subjects minimally clothed without shoes, using digital scales (Seca 707: range 0.1–150 kg) and recorded to the nearest 100 g. Height was measured in a standing position without shoes, using tape meter while shoulders were in a normal alignment. Waist circumference (WC) was measured at the umbilical level, and that of the hip at the maximum level. Measurements performed over light clothing, using an unstretched tape meter, without any pressure to body surface and measurements were recorded to the nearest 0.1 cm. Waist-to-hip ratio (WHpR) was calculated as waist circumference (WC) divided by hip circumference (HC) and WHtR was calculated as WC divided by height [16]. Body mass index (BMI) was calculated as weight in kilograms divided by height in meters squared. The standard 2-hour postchallenge plasma glucose (2h-PCPG) test, including fasting plasma glucose (FPG), was performed for all individuals older than 20 years of age, not on antidiabetic drugs. A blood sample was taken after 12–14 h overnight fasting and was centrifuged within 30–45 min of collection. All blood analyses were performed at the TLGS research laboratory on the day of blood collection. The standard 75 g 2-hours postchallenge plasma glucose (2h-PCPG) test was performed for individuals older than 20 years of age not on antidiabetic medication. Plasma glucose was measured by enzymatic colorimetric method using glucose oxidase kit (Pars Azmoon Inc., Tehran, Iran). Inter- and intra-assay coefficients of variations (CVs) at base line and during follow up examinations were all <2.2%. For lipid measurements, total cholesterol and triglyceride kits (Pars Azmoon Inc., Tehran, Iran) were used. Triglycerides (TGs) were assayed using enzymatic colorimetric assay with glycerol phosphate oxidase. HDL-cholesterol (HDL-C) was measured after precipitation of the apolipoprotein B containing lipoproteins with phosphotungstic acid. All samples were analyzed when internal quality control met the acceptable criteria. Inter- and intra-assay CVs were 2% and 0.5% for HDL-C and 1.6% and 0.6% for TGs, respectively [17, 18].

### 2.3. Definition of Terms

Positive family history of diabetes was defined as having at least one parent or sibling with diabetes. Participants were classified as having diabetes at the baseline or during followup if they met at least one of these criteria: FPG ≥ 7 mmol·L^−1^, or 2h-PCPG ≥ 11.1 mmol·L^−1^ or starting taking antidiabetic medication [19]. TG/HDL-C was calculated by dividing TG by HDL-C.

### 2.4. Statistics

D'Agostino's test for skewness and kurtosis, was used to test the null hypothesis that the population distribution from which the data sample is drawn has a Gaussian (normal) distribution [20]. 

### 2.5. Multivariable Models and Estimation of Diabetes Risk Function

Weibull proportional hazard regression models were developed for interval-censored survival data, since the precise date of developing diabetes could not be determined and the TLGS records provided only an interval for each diabetes diagnosis. Among variable known to predict diabetes, we used variables on which data are readily available, and are simple and low cost. We followed statistical guidelines with respect to the significance of association of a variable with incident diabetes but also considered scientific and qualitative judgment as well. For example, although 2h-PCPG was statistically significantly associated with risk of incident diabetes we did not include it in the final model, since we deemed it as being costly, time-consuming, not routinely available, and bothersome to both patients and clinician. Age was not associated with risk of incident diabetes but was kept because it is known to have strong effect on the risk of diabetes. Furthermore, age improved the calibration of the final model. 

### 2.6. Assessment of Model Performance

We used several criteria to compare the overall predictive values of alternative models.

*Goodness-of-Fit*. How effectively a model describes the outcome variable is referred to as its goodness-of-fit. Akaike information criterion (AIC) was used as a measure of model fit and informativeness indicating whether the addition of new factors to a base model provides better risk prediction than the base model alone, provided that all of the same individuals are being assessed by both models. Difference in AIC > 10 was considered to be significant [21]. Bayesian information criteria which is of the same performance was also calculated.

*Discrimination*. Discrimination is the ability of a prediction model to separate those who develop diabetes from those who do not and is quantified by the *C* statistic, which is equivalent to the area under a receiver operating characteristic (ROC) curve for binary dependent variables [22]. In the survival analysis, *C* statistic measures the probability that a randomly selected person who developed an event, at the certain specific time has a higher risk score than a randomly selected person who did not develop an event during the same, specific follow-up interval [23, 24]. For *C*-indices of different models 95% confidence intervals were estimated with bootstrap sampling. 

*Calibration*. Calibration, as phrased by Hosmer and Lemeshow, “describes how closely predicted probabilities agree numerically with actual outcomes [25–27].” We examined calibration implementing a test very similar to the Hosmer-Lemeshow test has been proposed by D'Agostino and Nam [25]. As suggested by D'Agostino and Nam, calibration Chisquare values greater than 20 (*P* < 0.01) suggest lack of adequate calibration [25]. 

### 2.7. Point Score System

We developed a point-score system for predicting 6-year risk of incident diabetes from *α*-coefficients for variables associated with incident diabetes in multivariate accelerated failure time models using methods described elsewhere [28, 29]. SBP was divided into 5 categories as follows: <120, 120–129, 130–139, 140–159, and 160 mmHg or more [28]. FPG was divided into 4 categories as follows: <5.0, 5.0–5.5, 5.6–6, and 6.1–6.9, mmol·L^−1^ (<90, 90–99, 100–109, and 110–125 mg·dL^−1^). WHtR was divided into 4 groups based on the cut-points for quartiles: <50, 50–59, 60–69, and 70% or more. High TG/HDL-C was defined as levels above 1.5 (3.5 based on conventional units). In addition to these arbitrary predetermined classifications, we tested the cut-point using multivariate restricted cubic splines Weibull proportional hazard regression models [30]. The cut-points remained essentially the same, we, thus, used the predetermined ones, which are more likely to seem familiar to users. 

### 2.8. Beta-Cell Age

We defined beta-cell age as chronological age of a person with the same predicted risk but all risk factors at the normal levels (i.e., WHtR 0.50, no family history of diabetes, Ln (TG/HDL-C) = 0.531, and FPG = 4.9 (mmol·L^−1^)). To do this, we entered the normal levels of risk factors (defined above) into the risk functions and then solved for age, leaving risk probability as an independent variable. This resulted in beta-cell age, to which the probability output from the updated TLGS' diabetes risk prediction functions for each participant were introduced. In keeping with the limitations of beta-cell age, beta-cell ages at the extremes are simply reported as <34 or >71 years.

We certify that all applicable institutional and governmental regulations concerning the ethical use of human volunteers were followed during this research. Informed written consent was obtained from all participants and the Ethical Committee of Research Institute for Endocrine Sciences approved this study. We set the statistical significance level at a two-tailed type I error of 0.05. All statistical analyses were performed using STATA version 11.0 (STATA, College Station, TX, USA).

## 3. Results

We followed 5,960 (3,438 women) participants of the TLGS for a median of 6.2 years. During this 36,275 person-year followup, we documented 369 incident cases of diabetes. The annual incidence rate of diabetes was 0.85 per 1000 person (95% CIs 0.77–0.94). 

**Figure 2:**
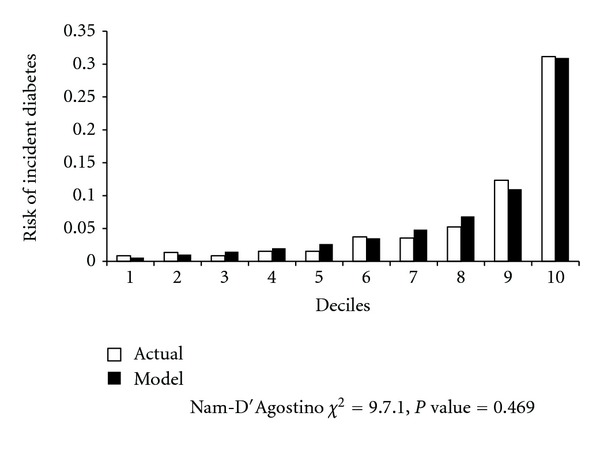
Six-year predictions for diabetes: performance measures based on the updated TLGS model.

Table 1 presents baseline characteristics of participants. The mean age of the participants was 42 years, ranging from 20 to 86 years.  Table 2 shows the contribution of the predictors to the risk of incident diabetes independent of each other. The model incorporated age, SBP, family history of diabetes, WHtR, TG/HDL-C, and FPG. Age did not significantly contribute to the risk of incident diabetes. We, however, maintained it in the model since it improved the fitness of the models. The updated TLGS' diabetes prediction model showed a good discrimination capacity as indicated by Harrell's *C* statistic of 0.830 (95% CIs 0.808–0.852). As the Figure 2 depicts, the model achieved a good calibration with Nam-D'Agostino *χ* ^2^ of 9.7.1 (*P* for lack of fitness = 0.469).

Table 3 shows how to utilize the point-score system for calculating a 6-year risk of incident diabetes. The highest weight was assigned to the FPG levels of 6.1–6.9 mmol·L^−1^. Risk of developing incident diabetes for individuals whose scores summed up to a point total of 14 or less was estimated to be less than 5 percent. However, more than 75% of cases of incident diabetes scored more than 14. Table 4 presents the predictive measures across continuum of the point totals. 

## 4. Discussion

The number of candles on our birthday cake may add up to our chronological age, but it doesn't necessarily equal our biological age: environmental factors, such as stress and diet, and genetics can speed up or slow down how the body ages. We, therefore, developed mathematical function by which to calculate a person's beta-cell age, a number that is intended to provide a more accurate and understandable assessment of a person's risk of developing diabetes. Combined with traditional risk-assessment measures, beta-cell age is a tool that can give clinicians a more precise understanding of a patient's risk, eliminating some of the uncertainty. It can help us identify and treat people before they develop diabetes. Just as important, is using beta-cell age as an important communication tool that helps clinicians better explain risk to their patients.

Six elements of the updated TLGS' diabetes prediction model contributed to the risk of incident diabetes were age, family history of diabetes, SBP, WHtR, TG/HDL-C, and FPG. Risk of developing diabetes is influenced by the length of exposure to risk factors that is poorly characterized by measurement of risk factors at one point in time [31]. Though, age may be a risk factor per se, it also serves as a representative of the length of exposure to risk factors [32]. This could explain why unaccompanied age is a powerful predictor of the future incident diabetes whereas, forcing age into the model already incorporating other risk factors attenuated the impact of age on the risk of incident of diabetes. 

Family history of diabetes has been shown to be a strong predictor of [11]. Family history of diabetes is not modifiable; notwithstanding, it can help communicate risk of developing diabetes. Some investigator argued that what is useful for a clinician to be an owl foreshadowing an inevitable risk. We feel, however, that having individuals with family history of diabetes informed of their risk of developing incident diabetes, could possibly render them motivated to attempt weight reduction so as to maintain their WHtR below 0.5. Theoretically, this approach may counterpoise the risk conferred by a family history of diabetes. Maintaining SBP levels below 120 mmHg can make up for the adverse effect of aging. Measures of abdominal obesity, as indirect indicators of visceral adiposity, have been extensively investigated with respect to the insulin resistance and diabetes [11]. WHtR of 0.5 has recently been recognized as a suitable global boundary value, this can give support to the simple public message “keep your waist circumference to less than half your height [33].” Hypertriglyceridemia has been considered as a risk factor for developing diabetes by American Diabetes Association [34]. Introducing TG and HDL-C as independent covariates in a regression model could possibly hinder capturing nirvana of predictability of them, owing to statistical multicollinearity and an intimate correlation between these variables in lipid metabolism [35]. Combining TG and HDL-C to TG/HDL-C ratio helped decreasing variance of estimates while retaining unbiasedness [11]. Insofar as diabetes is defined by the FPG levels, it is not surprising to observe FPG conferring the greatest risk for incident diabetes. We, however, documented that 70% of the cases of incident diabetes met criteria of 2h-PCPG ≥ 11.1 mmol·L^−1^. 

While choosing a model for prediction of diabetes the availability of risk factor data in the clinical setting, the optimal cut-point to define a positive test, and the simplicity of the model [13] must be considered. Some of previous studies are restricted with respect to age or sex [36, 37]; some used predictors like serum biomarkers, insulin resistance indices or surrogates, or genetic markers, which can only be measured by time-consuming, costly, or invasive testing procedures [38–40]. Complex models were shown to be unlikely to provide such increased predictive performances that justifies their complexity [40, 41]; this underscores the view that identification of adverse phenotypic characteristics remains the cornerstone of approaches for predicting the risk of diabetes [40–46].

In developing predicting rules, a trade-off between sensitivity and specificity, which depends on the threshold value (or cut-point) used to define a positive test result, is a necessity [47, 48]. It has been suggested [48] that in diabetes screening, tests with moderate sensitivity (about 60%) but high specificity (about 90%), repeated every 3 years, optimize the trade-off between disease detection and avoiding false-positive results [13]. If so, the “optimal” cut-point for the updated TLGS diabetes prediction model would be 15 (sensitivity: 61%, specificity: 84%). This point score corresponded to beta-cell age of 45 year. American Diabetes Association recommended that if no risk factor exist screening for diabetes should begin at 45 years of age [49]. However, in as much as health care system are unlikely to evaluate more than 25 percent of incident cases of diabetes, we would prefer to vote in favor of score of 10 where a predetermined sensitivity of 75% could be achieved. That is the fraction of population that needed subsequent testing would reduce to less than 25 percent.

The diabetes risk function of Table 2 is easily programmed, for example, as an Excel spreadsheet or as the score sheet of Table 3. In this study, we also present a new concept of beta-cell age. Here, the diabetes risk of an individual is transformed to the age of a person with the same risk but all other risk factors at the normal level. For example, a 40-year-old individual with risk factors above normal levels (WHtR 0.70, family history of diabetes, TG/HDL-C = 2, and FPG = 6.5 (mmol·L^−1^)) has the beta-cell age of a 71-year-old individual with normal risk factors.

Our finding needs to be interpreted in the context of its limitations. Except for those on antidiabetic drugs, cases of incident diabetes were defined on the basis of one measurement for FPG and 2h-PCPG, as is the case in almost all published data. Therefore the yield of model in terms of cases detected might be overestimated. The final sample may not be very representative of Iranians in general. It was an urban population and represents about 73% of the original sample. Physical activity and nutrition were not included in the models. They are important but can be very difficult to measure with adequate precision [28, 50]. Smoking and alcohol consumption are value-laden and so prone to underreporting [50]. Strengths of this lays in its performance as a large population-based cohort study that could reduce the selection bias, the use of measured (rather than reported) values for predictors and outcome, and the availability of determination of glucose levels in fresh venous blood in addition to using both FPG and 2h-PCPG for identifying diabetes. Finally, we took a comprehensive approach to model development using several statistics. 

## 5. Conclusion

In conclusion, using data from additional patients, we developed an updated version of the TLGS' diabetes prediction model which is still simple enough to allow calculating 6-year risk of diabetes with pencil and paper. Updated model achieved additional predictability as compared to the former version. Beta-cell age can provide a yardstick for patients to measure their risk of developing diabetes. Further research will be required to examine if such an approach could motivate individuals at risk to change their lifestyle more efficiently than could its currently available counterparts.

## Figures and Tables

**Table 1:** Baseline characteristics of participants.

Variable	Observations	Mean^a^	Minimum	Maximum
Age (years)	5960	41.98 (13.54)	20.00	86.00
Systolic blood pressure (mmHg)	5945	118.27 (17.60)	77.00	227.00
Diastolic blood pressure (mmHg)	5945	77.49 (10.50)	46.00	139.00
Waist circumference (cm)	5943	87.77 (11.82)	56.00	147.00
Height (cm)	5960	162.33 (9.02)	131.00	195.00
Cholesterol (mmol·L^−1^)	5960	5.39 (1.17)	2.20	14.79
Triglycerides (mmol·L^−1^)	5960	1.87 (1.20)	0.29	16.95
High-density lipoprotein cholesterol (mmol·L^−1^)	5958	1.09 (0.28)	0.36	2.85
Fasting plasma glucose (mmol·L^−1^)	5960	4.99 (0.54)	2.33	6.94
2-hour postchallenge plasma glucose (mmol·L^−1^)	5960	5.92 (1.63)	1.67	11.04
Assigned to life style modification interventions	5960	2206 (0.37)	—	—
Family history of diabetes	5960	1551 (0.26)	—	—

^a^Values for life style modification, interventions,and family history of diabetes are frequency (%).

**Table 2:** Multivariate model for predicting 6-year risk of incident diabetes.

Predictors	Hazard ratio	*P* value
Age (years)	1.01 (1.00–1.02)	0.031
Family history of diabetes	1.05 (1.03–1.06)	0.000
Waist-to-height ratio (%)	1.68 (1.36–2.08)	0.000
Triglyceride-to-high-density lipoprotein cholesterol ratio	1.07 (1.04–1.10)	0.000
Fasting plasma glucose (mmol·L^−1^)	5.39 (4.53–6.42)	0.000
Akaike information criteria	2798	
Bayesian information criteria	2845	
Hosmer-Lemeshow *χ* ^2^ (*P* value)	9.7 (0.462)	
Harrell's *C* statistic (95% CIs)	0.830 (0.808–0.852)	

**Table 3:** Implementing point-score system to estimate 6-year risk of incident diabetes.

Risk factor	Category	Points		Point total	Six-year risk of diabetes	Beta-cell age (years)
Age (years)	20–29	0		<6	<0.05	<34
	30–39	1		10	0.10	41
	40–49	1		13	0.16	41
	50–59	2		15	0.21	44
	60+	2		16	0.24	46
				17	0.28	50
WHtR (%)	<50	−2		18	0.32	49
	50–59	1		19	0.37	55
	60–69	4		20	0.42	54
	70 or greater	6		21	0.48	55
				22	0.54	62
Family history of diabetes	no	0		23	0.60	60
	yes	3		24	0.66	61
				25	0.72	64
TG/HDL-C	<1.5	−3		26	0.78	67
	1.5 or higher	6		≥27	≥0.80	≥71
Fasting plasma glucose (mmol·L^−1^)	<5.0	−5				
	5–5.5	4				
	5.6–6.0	9				
	6.1–6.9	14				

TG/HDL-C: triglyceride-to-high-density lipoprotein cholesterol ratio; WHtR: waist-to-height ratio.

**Table 4:** Diagnostic properties for incident diabetes of the TLGS' prediction function cut-offs.

	Diagnostic property (95% CIs)
Sensitivity	61.5 (56.3–66.5)
Specificity	84.0 (83.40–85.0)
Area under the receiver operating characteristic curve	0.73 (0.704–0.755)
Likelihood ratio positive	3.95 (3.57–4.37)
Likelihood ratio negative	0.46 (0.40–0.52)
Odds ratio	8.7 (6.9–10.8)
Positive predictive value	21.0 (18.4–23.3)
Negative predictive value	97.1 (96.6–97.5)
